# KrF Laser and Plasma Exposure of PDMS–Carbon Composite and Its Antibacterial Properties

**DOI:** 10.3390/ma15030839

**Published:** 2022-01-22

**Authors:** Dominik Fajstavr, Bára Frýdlová, Silvie Rimpelová, Nikola Slepičková Kasálková, Petr Sajdl, Václav Švorčík, Petr Slepička

**Affiliations:** 1 Department of Solid State Engineering, University of Chemistry and Technology Prague, 166 28 Prague, Czech Republic; dominik.fajstavr@vscht.cz (D.F.); bara.frydlova@vscht.cz (B.F.); nikola.kasalkova@vscht.cz (N.S.K.); vaclav.svorcik@vscht.cz (V.Š.); 2 Department of Biochemistry and Microbiology, University of Chemistry and Technology Prague, 166 28 Prague, Czech Republic; 3 Department of Power Engineering, University of Chemistry and Technology Prague, 166 28 Prague, Czech Republic; petr.sajdl@vscht.cz; 4 Faculty of Mechanical Engineering, J. E. Purkyne University, 400 96 Usti nad Labem, Czech Republic

**Keywords:** excimer laser treatment, plasma treatment, nanostructure, antibacterial properties, carbon nanotubes, polydimethylsiloxane, wrinkle-like patterns, *E. coli*, *S. epidermidis*, antimicrobial activity

## Abstract

A polydimethylsiloxane (PDMS) composite with multi-walled carbon nanotubes was successfully prepared. Composite foils were treated with both plasma and excimer laser, and changes in their physicochemical properties were determined in detail. Mainly changes in surface chemistry, wettability, and morphology were determined. The plasma treatment of PDMS complemented with subsequent heating led to the formation of a unique wrinkle-like pattern. The impact of different laser treatment conditions on the composite surface was determined. The morphology was determined by AFM and LCM techniques, while chemical changes and chemical surface mapping were studied with the EDS/EDX method. Selected activated polymer composites were used for the evaluation of antibacterial activity using Gram-positive (*Staphylococcus epidermidis*) and Gram-negative (*Escherichia coli*) bacteria. The antibacterial effect was achieved against *S. epidermidis* on pristine PDMS treated with 500 mJ of laser energy and PDMS-C nanocomposite treated with a lower laser fluence of 250 mJ. Silver deposition of PDMS foil increases significantly its antibacterial properties against *E. coli*, which is further enhanced by the carbon predeposition or high-energy laser treatment.

## 1. Introduction

Nanotube composites, in which nanotubes are dispersed in a soft matrix, typically a polymer, are recently very attractive for practical applications owing to their unique properties combining the advantages of both materials. Carbon nanotubes (CNTs) are long cylindrical structures composed of typically ordered carbon atoms (similar to fullerenes). According to the structure, they are classified as single-wall CNTs (SWCNTs), double-wall CNTs (DWCNTs), and multiwall CNTs (MWCNTs) [1]. They were first discovered in 1985 as a by-product of graphite vaporization. The current industrial synthesis uses many techniques, such as laser ablation, the arc method, chemical vapor deposition, or a solar furnace [2]. SWCNTs by definition have a single wall, their diameter is around 1 nm, and their length is in the micrometer scale. Spatially, it is a layer of graphite, one atom thick, called graphene, “rolled” into a seamless hollow cylinder [3] with the ends mostly “clogged”. SWCNTs are more flexible than MWCNTs but more complex to manufacture. Their properties vary according to the structural arrangement of the atoms in the lattice but are generally highly electrically and thermally conductive, thermally resistant, and tough. MWCNTs have, as the name implies, a larger number of concentric walls, and their size is between 2 and 10 nm in the inner diameter and 20–70 nm in the outer diameter; the length is about 50 µm. MWCNTs may also appear in the form of a single graphene layer rolled up like a coil. MWCNTs have similar properties as SWCNTs but are more fragile and, due to their significantly more complex structure, are more prone to the presence of manufacturing defects [4]. DWCNTs are a relatively newly defined class of bilayer nanotubes with an outer diameter of less than 2 nm and a length ranging from a few hundred nanometers to units of micrometers. They consist of two nanotubes embedded in each other. The difference between their diameters and chirality affects the mutual interactions and, thus, the properties of the final DWCNTs. Because the outer wall of CNTs can be subjected to various changes and modifications, DWCNTs have recently gained increased attention. For example, the interactions caused by the chirality of individual CNT walls are responsible for the formation of the metallic or semiconductor character of nanotubes [5]. At the same time, they also show the flexibility of SWCNTs and the electrical and thermal stability of MWCNTs [4].

Silicones are generally materials with advantageous properties, such as flexibility, biocompatibility, optical transparency, and easy and relatively inexpensive preparation. However, these features limit their use to a small number of applications. Therefore, it is advantageous to form polymer composites with other chemical additives and fillers that can potentially alter existing polymer matrices and introduce new properties [6]. Preferred sought modifications include mainly improvements in mechanical and thermal resistance, thermal stability, permeability, optical transmittance, and electrical and conductivity capabilities. To date, several studies have been conducted comparing the results of a large number of different impurities, such as metal oxides (titanium, zinc, silver), other polymers, calcium carbonate, and carbon nanoparticles [5]. These hybrid materials not only combine the properties of inorganic fillers and polymer matrices but also give rise to new ones. Traditional composite materials can exhibit local heterogeneity, which can lead to a loss of the desired capabilities. Therefore, it is important to disperse as much as possible so that the nanoparticles can interact with the polymer at the molecular level. Composite fillers can be referred to as nano when at least one of the particle dimensions corresponds to a nanometric scale [3,7].

Polymer composites with carbon nanoparticles naturally differ in their properties according to the type of silicone (polymer) used, but also according to the type of carbon nanoparticles, their representation, and spatial arrangement [8,9,10]. However, the carbon in admixtures improves the mechanical, thermal, and especially electrical conductivity properties of a material. CNT/polymer nanocomposites with their superhydrophobic surfaces can provide a substrate of desired optical, mechanical, thermal, and electrical properties [11,12,13,14,15,16]. Polydimethylsiloxane (PDMS) as a soft matrix is non-toxic, inert silicon-based organic polymer. A significant improvement in the mechanical strength of a composite is achieved right after polymerization and crosslinking [17,18]. The essential characteristic of the hydrophobic properties of the surface is nanoscale roughness according to multiscale morphology analysis [19]. Several strategies focusing on nanocomposite construction have been performed. One of them describes how a polymeric substrate can be prepared at the nanoparticle synthesis phase. Graphene oxide aerogels/PDMS were studied in the literature as well [20]. The materials exhibited significant water adhesion and compressibility due to their surface being shaped as foam-like structures. These structures are described in the study of Li et al. [21].

At present, perhaps the most frequent use of carbon nanocomposites is related to the modification of electrical conductivity. Silicones (especially the aforementioned PDMS) are biocompatible and together with nontoxic carbon (certain types of structures) form an ideal combination for various bodily applications [22]. Therefore, they are the subject of tissue engineering research, especially in the development of flexible and soft electronics, which can be incorporated directly into the human body. These are various sensors (motion, temperature, mechanical), flexible electronic microfluidics, and the so-called e-skin (i.e., prosthetic skin) [23]. Use in conventional electronics, such as touch screens or organic light-emitting diode (OLED) technologies, is usually not possible due to the lack of optical transparency of these composites.

The unique material properties of PDMS enable its use in a wide range of applications. In the liquid state, it is used in cosmetics (in shampoos and conditioners) and lubricants as well as in the food industry (as E900, an additive in frying oils preventing them from splashing). In the solid-state, it is then used in the form of coatings for its hydrophobic properties. However, its most significant use is mainly in medicine [24]. Silicones can be considered to be a group of synthetic polymers with the longest history of testing and investigation of biocompatibility in various medical applications, but especially in implants. Although biocompatibility and its definition are still the subjects of speculation, in vitro and in vivo studies have proven that silicones are indeed biocompatible [25]. Since they are used in a large number of forms (liquids, gels, elastomers), it is necessary to monitor possible additives. Even though the polymer alone does not elicit any bodily response, polymerization admixtures and catalysts can. However, many studies have confirmed both the biocompatibility and the biological resistance of silicone elastomers [26]. The only thing limiting their use is not entirely good mechanical properties, namely, mostly their low tensile strength, tendency to tear, and gradual abrasion. However, at present, PDMS is still utilized in many different forms. Since the 1940s, silicone coatings have been used to treat laboratory glass, needles, and syringes to prevent blood clotting. This was followed by their use in the first body implants, which are now an integral part of plastic surgery, and more recently, they have also been used for the production of contact lenses [27].

One of the methods of modifying prepared carbon-polymeric substrates and creating a surface with new physical or chemical properties is laser modification. Laser techniques working with a specific frequency and wavelength can modify a wide spectrum of substrates [28,29]. By laser modification, various surface structures with different shapes or new forms of elements can be prepared, such as linear, globular, and web structures [30,31]. The surface structure topography can be governed by many parameters, such as laser type, fluence, and pulse duration, as well as the number of pulses on the treated substrate and repetition frequency. Zhou et al. documented a study based on the influence on silicon substrates etched to form microgrooves with femtosecond and nanosecond laser [32]. The result of this study was that the microgrooves obtained with femtosecond lasers are smoother and have a smaller top width of a groove [33]. In other studies, antibacterial properties are achieved by the synergic effect of laser surface nanopatterning of a polymeric substrate and thin metallic layer deposition with subsequent laser irradiation [34]. Laser modification of the surface polymer enables direct control of cell response to the material. This phenomenon is desirable for constructing a biocompatible material or for enhancing its properties [35]. Material biocompatibility is closely related to surface roughness, which reflects surface topography created by laser modification, and it is an important factor for the preparation of an antibacterial substrate [36,37]. Therefore, an increase in the antibacterial effect and biocompatibility of the polymeric composites achieved by laser modification offers new attractive material properties for applications in the healthcare industry and medicine [38,39].

We applied a unique high-energy excimer laser for surface activation of a PDMS substrate. To our best knowledge, such excimer treatment in combination with exposure of carbon-based PDMS composites with carbon nanotubes has not been studied so far. As an alternative method for surface treatment, also plasma exposure combined with heat treatment was applied. PDMS substrates were either exposed to pristine or doped with different amounts of carbon multiwalled nanotubes. Surface physicochemical changes were studied by atomic force microscopy (AFM) and scanning electron microscopy. The surface chemistry was characterized by X-ray photoelectron spectroscopy and energy dispersive spectroscopy (EDS/EDX). The selected combination of surface treatment methods led to an increase in antibacterial properties for both Gram-positive and Gram-negative bacteria strains. Particularly, high-energy excimer exposure of PDMS and a PDMS composite significantly enhanced the antibacterial properties against the growth of *S. epidermidis*.

## 2. Materials and Methods

### 2.1. Materials

A pre-prepared 10 g kit for the preparation of PDMS films was purchased from Sigma-Aldrich (St. Louis, MO, USA) under the trade name Sylgard^®^ 184, registered by Dow Corning Corporation. The polymer matrix is vinyl-terminated polydimethylsiloxane. The PDMS composite prepared from this kit should reach a tensile strength (UTS) of about 5.2 MPa. Additionally, 50 μm thick PDMS elastomeric films were purchased from Goodfellow (UK) in the form of 150 × 200 mm sheets. According to the manufacturer, these colorless matt and flexible films are well gas permeable, thermally stable, and highly chemically and UV resistant.

Carbon nanofibers (CNF) were supplied (Sigma-Aldrich) as a fine black powder with a molar mass of 12.01 g·mol^−1^. The fibers are a product of Pyrograf^®^ Products Inc. prepared by the floating catalyst vapor-grown method. The carbon nanofibers thus prepared are hollow and, after heat treatment at 2900 °C, free of catalyst (iron) residues. The final iron content is reported to be less than 100 ppm. CNFs should be 100 nm in diameter, and their length should be in the range of 20–200 μm. The density is reported to be 1.9 g·mL^−1^ at 25 °C, and the melting point is found in the region of 3652–3697 °C. Toluene was chosen as the primary solvent since PDMS is miscible with it, and also because it is a relatively inexpensive and available solvent that does not require any special precautions. Another solvent chosen was anhydrous ethanol, which was used to make control samples for grafting PDMS films.

### 2.2. Foil Preparation

The solvent-casting method was chosen for the preparation of PDMS nanocomposite films. Carbon nanofibers were weighed into 10 mL glass vials according to preselected ratios to form films with different weight contents of CNFs. An amount of 5 mL of toluene was added to the preweighed nanofibers by an automatic pipette, and the suspension thus prepared was placed in a XUBA 1 ultrasonic bath (Grant, UK) for 1 h. PDMS was prepared by mixing a polymer matrix and a crosslinking agent at a ratio of 10:1 from a preweighed Sylgard^®^ 184 premix. Both liquids were mixed by hand in a plastic package without being opened for 10 min. The polymer mixture thus prepared was then weighed into vials with an already homogenized suspension of CNFs in toluene, and the whole was mixed by hand for another 5 min.

This liquid nanocomposite PDMS was then poured and shaken gently into a Petri dish (diameter of 10 cm) with a 50 μm thick backing fluorinated ethylene propylene film (GoodFellow GMBH, Hamburg, Germany). The liquid films thus prepared were then placed in a vacuum chamber for 10–15 min to remove bubbles and then transferred to an oven, where they were left at 100 °C for 2 h to polymerize. PDMS films with 1% and 2% CNFs by weight were prepared by this method. Besides, a control pristine film of Sylgard^®^ 184 was prepared, without the addition of carbon nanofibers. At the same time, a film with 2 wt.% CNFs was also prepared, with the only variation of the preparation process, not using an ultrasonic bath, but only manual mixing. The use of an ultrasonic bath has proven to be a crucial step in the formation of films with evenly distributed carbon nanofibers. The thickness of prepared foils was determined to be 670 ± 1.5 µm for PDMS and 630 ± 6.1 µm for PDMS with CNTs. The disproportion in thicknesses of these two was probably caused by the CNTs and changes in the viscosity of the solution. Due to the relatively high viscosity of PDMS (at room temperature), the CNFs were predispersed in toluene, thus forming a relatively stable solution that did not separate even after 3 weeks of observation. In contrast, before sonication, the phases were separated in the order of seconds. Ultrasound not only dispersed the nanofibers into the entire volume of the solvent but also helped segregate the resulting clusters of nanofibers.

### 2.3. Surface Modification

The prepared PDMS films containing carbon nanofibers were in the next part of the work exposed to several surface modifications and their combinations. Samples were modified with cold argon plasma. The modification was performed using a BAL-TEC Sputter Coater SCD 050 sputtering device, with the setting of the “etching” mode, at a pressure of about 10 Pa and outputs of about 3 W (5 mA, 600 V) and 8 W (11 mA, 730 V) for times of 80, 240, and 480 s. After plasma modification, selected samples were further thermally stressed by placing them in an oven at 100 °C for 1 h.

PDMS elastomeric films purchased from Goodfellow (UK) in the form of 150 × 200 mm^2^ sheets were used for the study of the effect of subsequent carbon/silver deposition and heat treatment. The pristine PDMS samples were covered with carbon layers as the primary layer before Ag deposition. Carbon layers were deposited using an SCD 050 carbon thread evaporation device by the flash evaporation process, using a carbon filament (Leica). The carbon was then deposited with a current of 1.5 A (pressure of 4 Pa). The polymer substrate was at a distance of 5 cm (C5) from the carbon filament. Corresponding thicknesses of carbon layers were determined on a quartz glass substrate with a scratch method and were determined with an atomic force microscope. A distance of 5 cm represents a thickness of 8.1 ± 0.7. Silver layers were subsequently deposited with Quorum Q300T ES, and different sputtering currents ranging from 10 to 80 mA were used with deposition times of 300 or 500 s.

Surface properties were also modified with a high energy pulsed excimer KrF laser (Coherent Inc., Leap 100K, Santa Clara, CA, USA) with a wavelength of 248 nm, a pulse duration of 20–40 ns, and a repetition rate of 10 Hz, with a stabilized maximum energy range of 900–1000 mJ and beam dimensions of 32 × 13 mm^2^. An aperture of 30 × 10 mm^2^ was used in the experiments.

### 2.4. Analytical Methods

The surface morphology of the substrates was analyzed using an atomic force microscope, Dimension Icon (Bruker Corp., Billerica, MA, USA). QNM mode in the air was used for the analysis with a silicon tip on Nitride Lever SCANASYST-AIR and a spring constant of 0.4 N·m^−1^. The measured data were processed with the NanoScope Analysis software.

The wettability of the studied material surface was detected by the determination of water contact angle (CA). Analysis of CA was realized at room temperature with distilled water at six different positions of 3 samples using the surface energy evaluation system (Advex Instruments, Brno, Czech Republic) with automatic pipetting and a drop volume of 8.0 ± 0.2 μL. The method error was ±5%.

As a complementary technique for surface morphology characterization, we applied the scanning electron microscope LYRA3 GMU (Tescan, Brno, Czech Republic). The acceleration voltage was set to 10 kV. Metallization of samples was performed by a sputtering technique (Quorum Q300T) by deposition of a Pt conductive layer (thickness of 20 nm, Pt target, purity of 99.9995%). The elemental composition was measured by energy-dispersive X-ray spectroscopy (EDS, analyzer X-MaxN, 20 mm^2^ SDD detector, Oxford Instruments, United Kingdom). The accelerating voltage for SEM-EDS measurement was 10 kV.

The elemental composition of the surface of prepared and exposed samples was analyzed by X-ray photoelectron spectroscopy (XPS) by use of a spectrometer, ESCAProbeP (Omicron Nanotechnology Ltd., Taunusstein, Germany). Elemental concentrations were determined from the individual peak areas using CasaXPS software. The spectra were acquired using monochromatic X-ray radiation (1486.7 eV) and very low energy of charge compensating electrons.

### 2.5. Antibacterial Study

The antimicrobial activities of the prepared materials were evaluated using two types of model bacterial strains (i.e., *S. epidermidis* (DBM 3179, Gram-positive bacteria) and *E. coli* (DMB 3138, Gram-negative bacteria)). Each type of bacteria was used for inoculum preparation by the following procedure: from agar plates of the stock bacteria, one colony-forming unit (CFU) was inoculated into liquid Luria-Bertani medium, which was followed by 16 h cultivation at 37 °C while gently shaking. Then, the optical densities of the bacterial inocula were determined at 600 nm, after which they were serially diluted. *E. coli* and *S. epidermidis* were inoculated at amounts of 2.10^−4^ and 4.10^−4^ of CFU, respectively, per 1 mL of phosphate-buffered saline, into which the evaluated samples were immersed. Then, the samples were very slightly mixed and incubated (dynamically) for 4 h at laboratory temperature. Next, the samples were again mixed, and 25 μL drops of each sample (5 replicates) were pipetted onto Luria-Bertani and plate count agar plates (*E. coli* and *S. epidermidis*, respectively). The agar plates were incubated for 16 h at 37 °C, after which the CFU number was counted on each plate and compared with controls (bacteria on agar plates from samples incubated without the evaluated samples, i.e., only phosphate-buffered saline for the same period). The experiments were performed in sterile conditions.

## 3. Results

### 3.1. Surface Morphology of Plasma-Treated Samples

**Figure 1:**
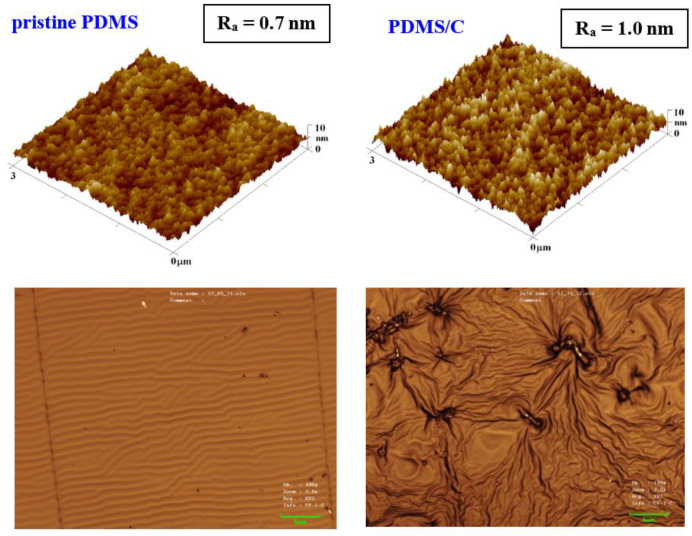
Surface morphology of a pristine PDMS foil and PDMS with 2 wt.% of carbon nanotubes (PDMS/C) in the upper part of the image; bottom images represent laser confocal microscopy scans of the same samples subsequently treated by plasma at 8 W for 240 s (a selected area of 43 × 32 μm^2^).

The first step of this work was to characterize the pristine PDMS foil; therefore, we focused on the surface morphology of a prepared pristine PDMS polymer. The morphology analysis was complemented with the analysis of PDMS with 2 wt.% of CNTs (further noted as PDMS/C), as is introduced in Figure 1. It can be concluded that the introduction of CNTs induced a slight increase in surface roughness (0.7 vs. 1.0 nm), as is obvious from the first line in Figure 1. The same pattern was followed for larger areas. Based on our previous experience with heat and plasma treatment of either pristine, doped, or plasma-modified polymer foils [40], we proceeded with the aforementioned surface treatments accompanied also by high-energy excimer laser treatment. The preliminary testing of plasma exposure of either a pristine or a carbon composite PDMS foil revealed the wrinkle-like formation, the unique pattern previously acquired (e.g., for PLLA biopolymer subsequently sputtered with noble metals or carbon), which underwent a heat treatment procedure [40]. The major difference in surface properties was found in the comparison of surface morphology of pristine PDMS and a PDMS-C composite, which was realized with an AFM and laser confocal microscope (LCM). The former one, when treated by plasma at 8 W and 240 s, exhibited the formation of a relatively narrow ripple-like pattern, often found on laser-treated aromatic polymers, such as PEN or PS [30,38] (Figure 1, bottom line). On the contrary, the PDMS-C composite that underwent the same activation procedure exhibited a wrinkle-like pattern, which was less homogeneous, but higher roughness and effective surface area of the pattern were achieved. The first step of plasma activation of both pristine and PDMS-C polymer foils was followed by heat treatment.

**Figure 2:**
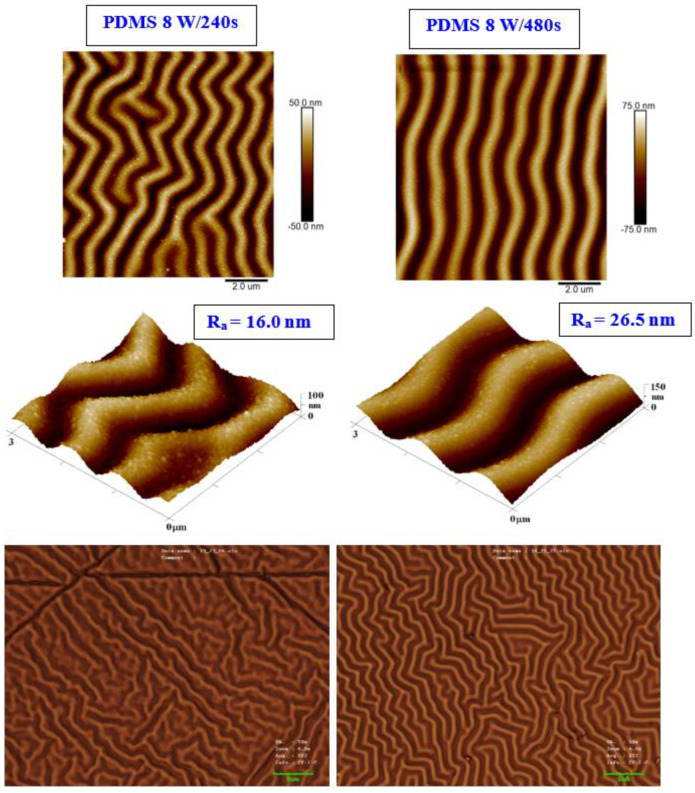
The surface morphology of a PDMS foil was treated by plasma at 8 W for 240 and 480 s and subsequently heated for 1 h at 100 °C. The first line represents the 2D morphology of samples with an inspected area of 10 × 10 μm^2^; the second line represents a 3D detail of the sample images (3 × 3 μm^2^). The bottom line represents laser confocal microscopy scans of the same samples with an inspected area of 43 × 32 μm^2^.

We present an analysis of a heat-treated and plasma-treated PDMS foil with an exposure power of 8 W and 240 s, which exhibited the most pronounced surface changes. As it is obvious from Figure 2, the simple technique of heat treatment had a significant effect on the formation of a wrinkle-like pattern. The higher was the plasma power applied, the more pronounced the wrinkle-like pattern was created, which was confirmed both by AFM and LCM analyses (see Figure 2). The appearance of a wrinkle-like pattern is connected to the formation of a polymer bilayer, one with an increase in the amount of the crystalline phase (as the amorphous phase is revealed by plasma ablation), the effect previously described for PLLA-metal bilayers, heated plasma-treated PLLA, or PLLA–carbon composites [40].

As it is obvious from detailed AFM images, on the wrinkled surface there appeared also granular nanostructures, which were present both on plasma-exposed and subsequently heated samples. As it is obvious from both AFM and LCM images presented in Figure 2, the sample exposed to plasma for a longer period of 480 s (at 8 W) exhibited a more regular wrinkle-like pattern compared with that exposed to 240 s. This is probably connected to a different amount of the amorphous phase ablated from the PDMS surface during the plasma activation process. Even though the plasma treatment induced a more regular pattern, its height increased, and thus the average surface roughness increased as well (from 16.0 to 26.5 nm).

**Figure 3:**
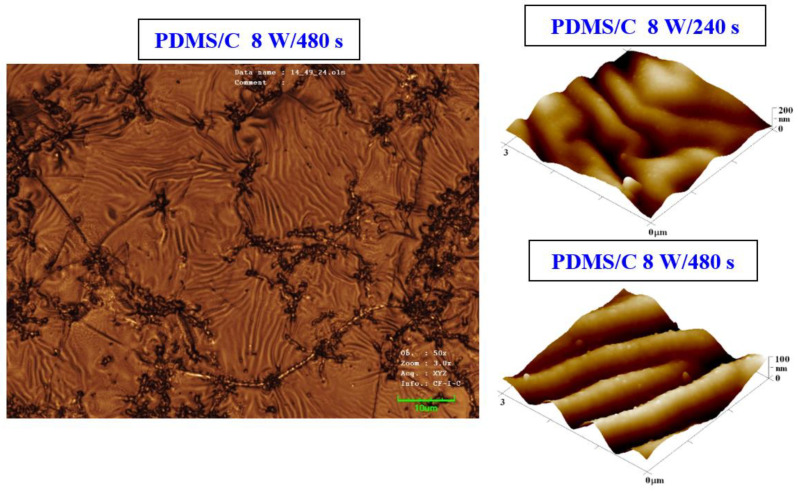
Image of the surface of a PDMS/C foil (2 wt.% of C) after plasma modification (8 W, 480 s) and thermal stress (1 h, 100 °C) taken by a confocal microscope with a displayed area of 85 × 64 μm^2^ (left image). AFM images of plasma-modified and thermally stressed (1 h, 100 °C) composite films C (PDMS/C 2 wt.%); the sample was modified by 8 W for 240 s (R_a_ = 27.7 nm) and by 8 W for 480 s (R_a_ = 18.1 nm).

The procedure of heat treatment was also applied to samples that were doped with 2 wt.% of CNTs. The argon plasma pretreatment was used only as of the process of activation of a primary substrate, before deposition of a carbon layer and/or silver layer by the consecutive sputtering process. The PDMS–carbon nanocomposite also underwent plasma treatment, and the resulting changes in surface morphology are presented in Figure 3. We also found that on the PDMS-C nanocomposite, which was exposed to plasma and subsequently heat-treated, the wrinkle pattern appeared (Figure 3). The formation of wrinkles was less homogeneous, and the effect of a long time of plasma exposure leading to a pattern with higher homogeneity can be observed as in the case of pristine PDMS. Images from LCM revealed some inhomogeneous clusters, which were formed during pattern formation (Figure 3, left image) on the larger inspected area.

### 3.2. Surface Chemistry of Plasma-Treated Samples

**Figure 4:**
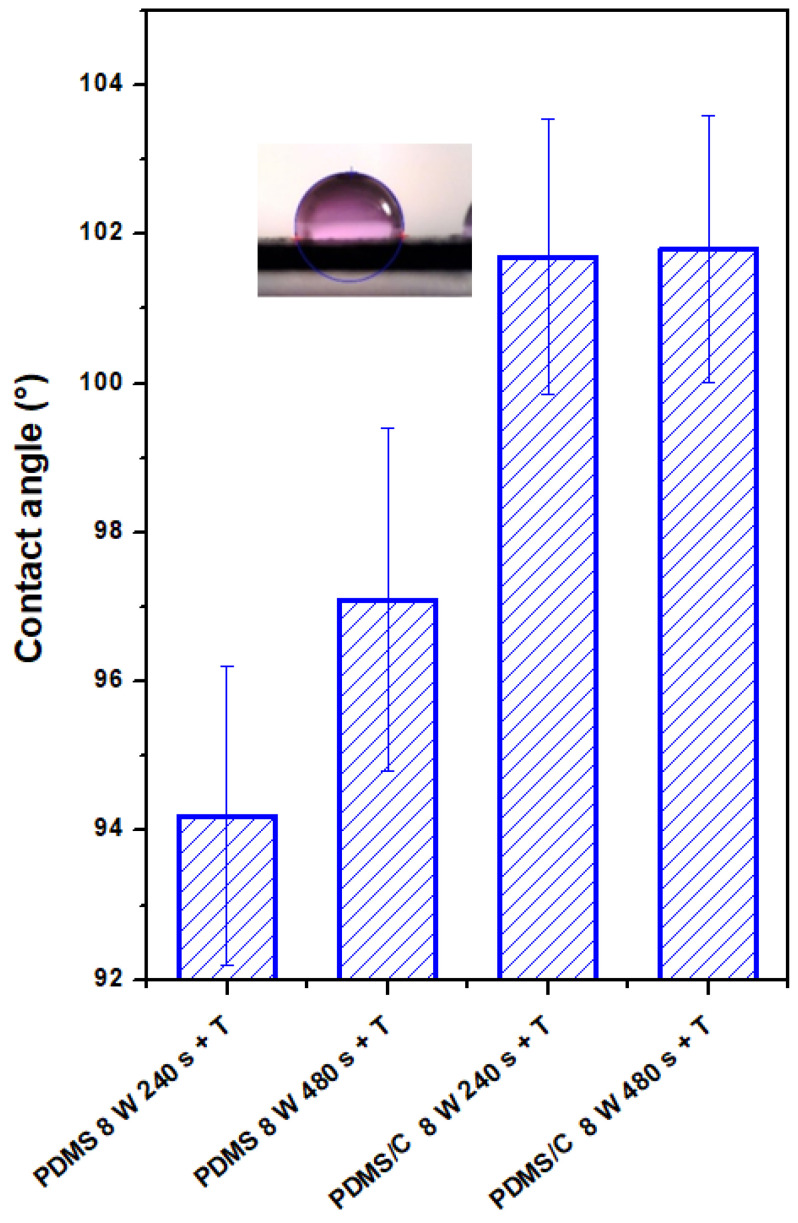
Contact angles of wettability of plasma-modified and thermally stressed PDMS films, two different exposure powers (3 and 8 W), and exposure times (240 and 480 s) were used.

The plasma-treated PDMS and PDMS/C samples, which were subsequently heat-treated, were studied from the point of changes in their surface wettability and chemistry. The CA of the pristine PDMS sample was determined to be 102.0 ± 1.2°. As it is obvious from Figure 4, two major phenomena can be found if we compare the wettability of plasma-treated samples with different exposure times and subsequently heated samples. As it is obvious, there is a difference in CAs for plasma-treated samples under certain conditions (8 W, 240, and 480 s) and the same exposure conditions conducted on PDMS-C composites. Heat treatment, which induces a wrinkle/ripple-like pattern on a plasma-treated PDMS surface, induces stabilization of CAs at different values, and higher values were observed for the longer exposure time (480 s). On the contrary, the wettability of heated PDMS-C composites was not influenced significantly by previously applied plasma treatment and the exposure time of treatment, with stabilized CA at approximately 101.5°.

**Figure 5:**
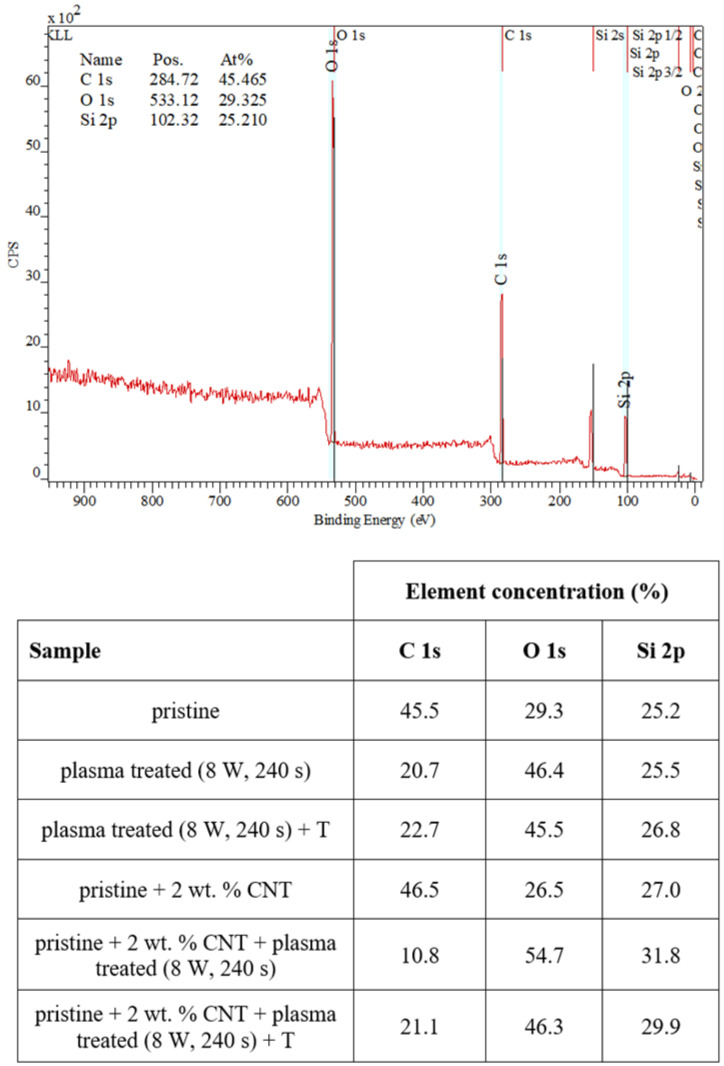
XPS spectrum of a pristine PDMS foil and changes in concentration of the monitored elements on measured pristine, doped, plasma-treated (8 W and 240 s), and heated PDMS samples (100 °C, 1 h).

We also examined the surface chemistry so that we can support our conclusions regarding surface changes of plasma-treated or heated samples. Figure 5 shows a spectrum of pristine PDMS and corresponding changes in surface chemistry. In Figure 5, it is evident that the simple addition of CNTs into the PDMS matrix-induced only slight changes in carbon concentration (an increase) and surface oxygen concentration (a decrease by approximately 1 at.% of carbon). The major effect of surface chemistry changes was observed after plasma treatment. The major increase in surface oxygen concentration was observed for both pristine and PDMS-C samples, which were treated with argon plasma. The PDMS samples showed an even higher increase in oxygen concentration after plasma exposure at 8 W and 240 s, and the concentration increased above 50 at.%. Heat treatment usually induces a decrease in oxygen concentration and a major increase in CA, which was observed, for example, for polymethyl pentene [41]. In Figure 5, we can see that the subsequent heat treatment induced only a mild decrease in oxygen concentration, and even stabilized samples had a significantly large oxygen concentration compared with nontreated samples.

### 3.3. High-Energy Excimer Treatment

**Figure 6:**
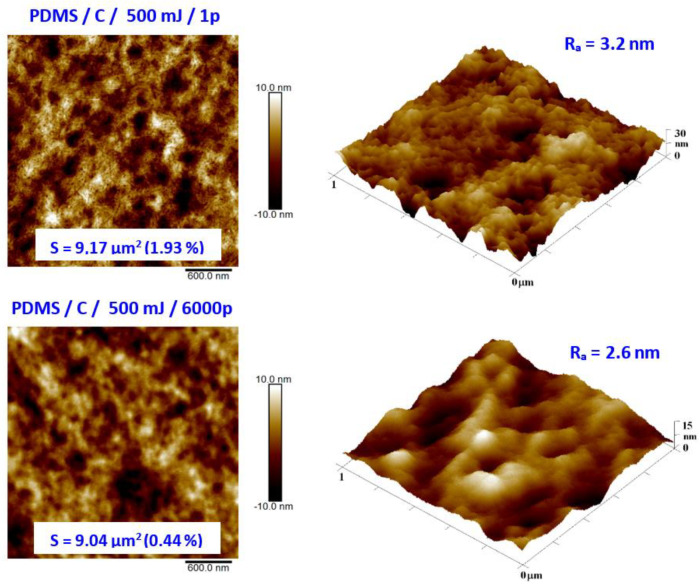
Surface morphology of PDMS-C film treated with high-energy excimer laser with a laser energy of 500 mJ and numbers of pulses of 1 and 6000. The selected image areas are 3 × 3 μm^2^ (2D) and 1 × 1 μm^2^ (3D). Effective surface area (S) and average surface roughness (R_a_) is introduced.

As the second major surface activation, we examined in detail the surface physicochemical changes induced on the PDMS surface by a high-energy excimer laser. We aimed at both surface morphology and surface chemistry changes. Pristine PDMS and PDMS-C nanocomposite morphology and the influence of high-energy excimer exposure were examined in detail. The surface morphology of a high-energy excimer laser is shown in Figure 6. We chose the particular samples treated only with one shot of 500 mJ and compared it with treatment with the same laser energy but several pulses (6000). As it is obvious from AFM analysis, the PDMS surface morphology was altered. As it is obvious from Figure 6, the laser treatment leads to more pronounced surface ablation, which is accompanied by a diminishing PDMS surface nanopattern and a slight decrease in surface roughness. The higher was the number of laser pulses, the larger changes on the PDMS surface were observed (Figure 6).

**Figure 7:**
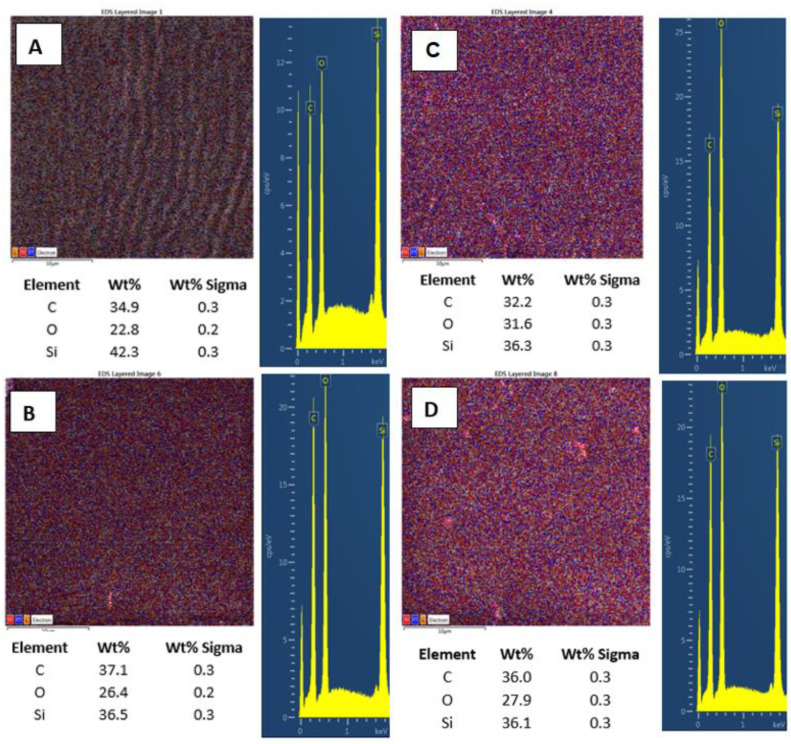
EDS/EDX analysis of PDMS (**A**), PDMS/C (carbon composite 2 wt.%) (**B**), PDMS laser-treated with 500 mJ and 6000 pulses, (**C**) and PDMS/C laser-treated with 500 mJ and 6000 pulses (**D**). Elemental mapping, weight concentration in %, and relevant EDS/EDX spectra are presented.

Further, we also monitored the changes in surface chemistry, acquired by EDS/EDX analysis, and the major effect of high energy exposure can be considered a significant increase in oxygen surface concentration (Figure 7A,B), from 22.8 to 31.6 at.%., while only a mild change in surface morphology is observed (on a large-scale area). The same pattern is followed for samples doped with 2 wt.% of CNTs. Compared with pristine PDMS, we determined an increase in carbon concentration of about 2.5%. Even though this increase was not as significant as we expected, it confirms the presence of CNTs homogeneously distributed in the PDMS matrix. As it is obvious from Figure 7C,D, the laser treatment of PDMS with CNTs also induced an increase in surface oxygen concentration; however, the change was only 1.5 wt.%, and the PDMS-C composite was less oxidized compared with pristine PDMS.

### 3.4. Antibacterial Properties

**Figure 8:**
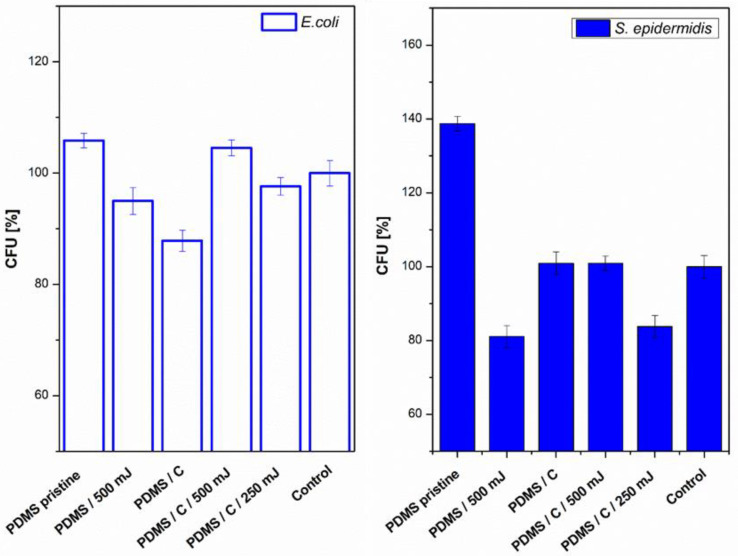
Antibacterial activity of polydimethylsiloxane (PDMS) samples against bacterial strains of *S. epidermidis* and *E. coli* after 4 h incubation. Samples: pristine PDMS, PDMS treated with high-energy laser (PDMS 500 mJ), PDMS composite with carbon (2 wt.%; PDMS/C), PDMS-C composite treated with 500 and 250 mJ, and control (bacteria in physiological solution). CFU represents colony-forming units grown on agar plates.

Next, we also determined the antibacterial properties of both pristine PDMS and PDMS–carbon composites together with the effect of a high-energy excimer laser. The antibacterial activity of the samples was evaluated against two bacterial strains of *S. epidermidis* (Gram-positive bacteria) and *E. coli* (Gram-negative bacteria). As it is obvious from Figure 8, the highest antibacterial effect was achieved against *S. epidermidis* on pristine PDMS treated with an energy of 500 mJ and PDMS-C nanocomposite treated with a lower energy of 250 mJ. Additionally, for 500 mJ treatment, a considerable decrease in the number of colony-forming units (CFU) was detected. In the case of *E. coli,* the strongest antibacterial effect was determined only for a simple PDMS-C composite without additional laser activation.

**Figure 9:**
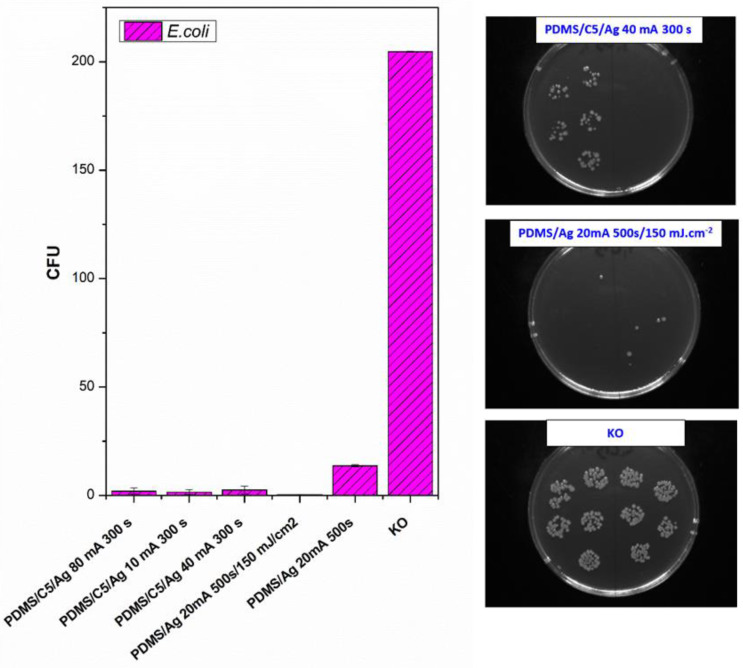
Antibacterial activity of polydimethylsiloxane (PDMS) samples (Goodfellow) against bacterial strain *E. coli* after 4 h incubation. Samples: PDMS coated with carbon and subsequently different thickness of Ag (PDMS/C5/Ag 80 mA 300 s; PDMS/C5/Ag 10 mA 300 s; PDMS/C5/Ag 40 mA 300 s), PDMS coated with carbon, and Ag and subsequently treated with high-energy laser (PDMS/Ag 20 mA 500 s/150 mJ·cm^−2^), PDMS coated only with Ag (PDMS/Ag 20 mA 500 s and control (bacteria in physiological solution)). Representative photos of CFUs on selected samples are introduced on the right side of the figure. CFU represents colony-forming units grown on agar plates.

As a consequent step, we followed a study of antibacterial properties involving pristine PDMS (Goodfellow) samples sputtered with Ag, the PDMS samples first covered with a thin carbon layer, followed by silver deposition, and finally, a combination of carbon and silver, which was subsequently treated with a high-energy excimer laser. The samples’ response to *E. coli* was studied. As it is obvious from Figure 9, the deposition of silver significantly enhances antibacterial PDMS properties, but carbon pretreatment or excimer exposure has a remarkable amplification antibacterial effect. As it is obvious from Figure 9, the carbon base layer improved the antibacterial properties of PDMS significantly, for samples that are subsequently covered with silver. If we look closely, a relatively large amount of silver (PDMS/Ag 20 mA 500 s) induced a strong antibacterial effect against *E. coli*, compared with the control sample, and the surface may be called antibacterial. However, when we deposited even a thinner layer of Ag onto PDMS predeposited with a thin carbon layer, which is a very economically feasible and facile procedure, the antibacterial effect was significantly amplified. “Antibacterial jackpot” is achieved if we combine the silver deposition with one-shot high-energy laser annealing. The selected photographs of corresponding samples are introduced in Figure 9. The possible mechanism of improved antibacterial performance mainly arises from the surface chemistry changes, particularly connected also to surface morphology. During laser treatment, the oxygen concentration on PDMS increases (Figure 7) compared with pristine PDMS, which has a positive effect on the antibacterial properties (Figure 8). Even the effect was achieved mostly for *S. epidermidis* and is not very pronounced. The silver deposition increases the antibacterial effect strongly since in a solution Ag^+^ ions are released, which promotes the antibacterial effect. The antibacterial silver properties can be enhanced by two procedures. The carbon predeposition is the first procedure, but more importantly, the following high-energy excimer exposure, as it is well visible in Figure 9. The effect of laser exposure is connected not only to an increase in oxygen concentration (subsequent oxidation of silver) but also to the globular formation, as was described in [42]. The combination of those two effects led to the formation of a surface with excellent antibacterial properties since an increased surface area subsequently promotes the Ag^+^ release.

**Figure 10:**
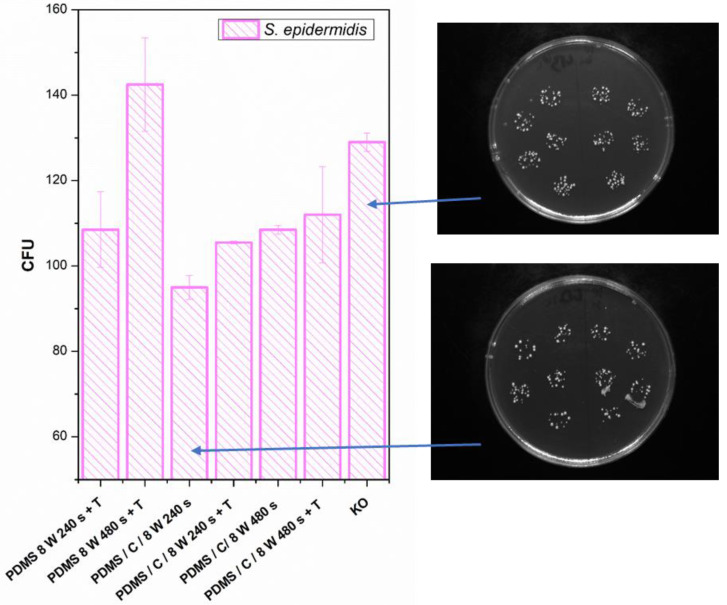
Antibacterial activity of polydimethylsiloxane (PDMS) samples against bacterial strains of *S. epidermidis* after 4 h incubation. Samples: plasma-treated PDMS with 8 W for 240 and 480 s with subsequent heat treatment (PDMS 8 W 240 s + T; PDMS 8 W 240 s + T); PDMS composite with carbon (2 wt.%) treated with plasma 8 W for 240 s (PDMS/C/8W 240s) and the same sample subsequently heat-treated (PDMS/C/8W 240s + T); PDMS composite with carbon (2 wt.%) treated with plasma 8 W for 480 s (PDMS/C/8W 480s) and the same sample subsequently heat-treated (PDMS/C/8W 480s + T); and control (bacteria in physiological solution). CFU represents colony-forming units grown on agar plates. Selected photos of samples are also introduced.

Finally, we determined the effect of plasma treatment or its combination on the antibacterial properties of either PDMS or its carbon composite. The antibacterial properties and selected photos of colonies on studied samples are introduced in Figure 10. We analyzed the antibacterial effect against both *S. epidermidis* and *E. coli*. The antibacterial properties against *E. coli* were similar or slightly weaker compared with *S. epidermidis*, so for the sake of clarity, we introduced only *S. epidermidis* results.

## 4. Conclusions

We successfully prepared homogeneous PDMS–carbon composites, which were subsequently treated with plasma or a high-energy excimer laser. PDMS treated by plasma at 8 W and 240 s exhibited the formation of a relatively narrow ripple-like pattern, often found on laser-treated aromatic polymers. The PDMS-C composite, which underwent the same activation procedure, exhibited a wrinkle-like pattern, which was less homogeneous than in the case of PDMS without carbon. A simple technique of material heat treatment had a significant effect on a wrinkle-like pattern formation. The higher was the plasma power applied, the more pronounced the wrinkle-like pattern was created, which was confirmed both by AFM and LCM analyses. The wrinkled surface also exhibited the granular nanostructure. Additionally, on the PDMS-C nanocomposite, which was exposed to plasma and subsequently heat-treated, the wrinkle-like pattern appeared, and the effect of a longer period of plasma exposure leading to a pattern with higher homogeneity can be observed as in the case of pristine PDMS.

Heat treatment, which induces the formation of a wrinkle/ripple-like pattern on a plasma-treated PDMS surface, leads to CA stabilization. The simple addition of CNTs into the PDMS matrix-induced only slight changes in carbon concentration. A major increase in surface oxygen concentration was observed for both pristine and PDMS-C samples. The laser treatment led to more pronounced surface ablation, which was accompanied by a diminishing PDMS surface nanopattern and a slight decrease in surface roughness. The major effect of high energy exposure can be considered a significant increase in oxygen surface concentration. Antibacterial studies revealed that the most pronounced antibacterial activity against *S. epidermidis* exhibited pristine PDMS treated with 500 mJ of laser energy and PDMS-C nanocomposite treated with a lower laser fluence of 250 mJ.

A silver nanolayer on a pristine PDMS foil increases its antibacterial properties against *E. coli*, which is further significantly enhanced by the carbon predeposition or high-energy laser treatment.

## Data Availability

All data contained within the article.
